# Pharmacological validation of dihydrofolate reductase as a drug target in *Mycobacterium abscessus*

**DOI:** 10.1128/aac.00717-23

**Published:** 2023-11-29

**Authors:** Wassihun Wedajo Aragaw, Dereje A. Negatu, Christopher J. Bungard, Véronique A. Dartois, Abdellatif El Marrouni, Elliott B. Nickbarg, David B. Olsen, Ralf Warrass, Thomas Dick

**Affiliations:** 1 Center for Discovery and Innovation, Hackensack Meridian Health, Nutley, New Jersey, USA; 2 Merck & Co., Inc., West Point, Pennsylvania, USA; 3 Department of Medical Sciences, Hackensack Meridian School of Medicine, Nutley, New Jersey, USA; 4 Merck & Co., Inc., Boston, Massachusetts, USA; 5 MSD Animal Health Innovation GmbH, Zur Propstei, Schwabenheim, Germany; 6 Department of Microbiology and Immunology, Georgetown University, Washington, USA; Bill & Melinda Gates Medical Research Institute, Cambridge, Massachusetts, USA

**Keywords:** non-tuberculous mycobacteria, NTM, folate pathway, synergy, DHFR, DHPS, ThyA

## Abstract

The *Mycobacterium abscessus* drug development pipeline is poorly populated, with particularly few validated target-lead couples to initiate *de novo* drug discovery. Trimethoprim, an inhibitor of dihydrofolate reductase (DHFR) used for the treatment of a range of bacterial infections, is not active against *M. abscessus*. Thus, evidence that *M. abscessus* DHFR is vulnerable to pharmacological intervention with a small molecule inhibitor is lacking. Here, we show that the pyrrolo-quinazoline PQD-1, previously identified as a DHFR inhibitor active against *Mycobacterium tuberculosis*, exerts whole cell activity against *M. abscessus*. Enzyme inhibition studies showed that PQD-1, in contrast to trimethoprim, is a potent inhibitor of *M. abscessus* DHFR and over-expression of DHFR causes resistance to PQD-1, providing biochemical and genetic evidence that DHFR is a vulnerable target and mediates PQD-1’s growth inhibitory activity in *M. abscessus*. As observed in *M. tuberculosis*, PQD-1 resistant mutations mapped to the folate pathway enzyme thymidylate synthase (TYMS) ThyA. Like trimethoprim in other bacteria, PQD-1 synergizes with the dihydropteroate synthase (DHPS) inhibitor sulfamethoxazole (SMX), offering an opportunity to exploit the successful dual inhibition of the folate pathway and develop similarly potent combinations against *M. abscessus*. PQD-1 is active against subspecies of *M. abscessus* and a panel of clinical isolates, providing epidemiological validation of the target-lead couple. Leveraging a series of PQD-1 analogs, we have demonstrated a dynamic structure-activity relationship (SAR). Collectively, the results identify *M. abscessus* DHFR as an attractive target and PQD-1 as a chemical starting point for the discovery of novel drugs and drug combinations that target the folate pathway in *M. abscessus*.

## INTRODUCTION

Treatment of lung disease caused by the opportunistic pathogen *Mycobacterium abscessus* delivers unsatisfactory cure rates despite long-term use of multidrug regimens with a macrolide, clarithromycin (CLR) or azithromycin, as the cornerstone (1). Treatment of *M. abscessus* is further complicated by the fact that the three subspecies, namely, *M. abscessus* subsp. *abscessus*, *M. abscessus* subsp. *massiliense*, and *M. abscessus* subsp. *bolletii*, present with differential drug susceptibility (2). The *M. abscessus* drug discovery pipeline is thin, and there is a need for novel target-lead couples to populate the early preclinical space (3).

Dihydrofolate reductase (DHFR) is a ubiquitous enzyme that catalyzes the NADPH-dependent conversion of dihydrofolate to tetrahydrofolate, involved in subsequent metabolic reactions such as thymidylate and purine nucleotide biosynthesis. DHFR is exploited as a target for anti-bacterial, anti-parasitic, anti-fungal, and anti-cancer drugs (4). Cotrimoxazole (Bactrim), the synergistic combination of DHFR inhibitors trimethoprim and sulfamethoxazole (SMX), with the latter targeting dihydropteroate synthase [(DHPS) the first enzyme in the folate pathway], is used for the treatment of a range of Gram-negative and Gram-positive bacterial infections (5). However, there are no DHFR inhibitors in clinical use against infections caused by mycobacteria. Interestingly, cotrimoxazole is recommended for the treatment of *M. abscessus* lung disease (1, 6) although the combination shows limited if any activity *in vitro* (7 – 9).

DHFR is encoded by *dfrA* (MAB_3090 c) in *M. abscessus* and is genetically essential, i.e., loss-of-function mutations due to transposon insertion prevent growth (10). However, evidence that pharmacological inhibition of DHFR with a small molecule results in effective inhibition of growth is lacking. Here, our objective was to determine whether *M. abscessus* DHFR is a vulnerable target and to identify a starting point for drug discovery.

We have previously shown that collections of anti-tuberculosis compounds provide a rich source for the identification of *M. abscessus* actives (11 – 13). Several screening campaigns yielded attractive hits against *M. tuberculosis* DHFR (14 – 18), including the pyrrolo-quinazoline PQD-1 with an MIC of 0.5 µM (19). Here, we show that PQD-1 also displays attractive potency against *M. abscessus*. We use PQD-1 as a tool compound to carry out biochemical, *in silico* docking, genetic, synergy, and structure-activity relationship (SAR) studies and to demonstrate activity against the subspecies of *M. abscessus* and a panel of clinical isolates.

## RESULTS AND DISCUSSION

### PQD-1 is active against *M. abscessus*

**Fig 1:**
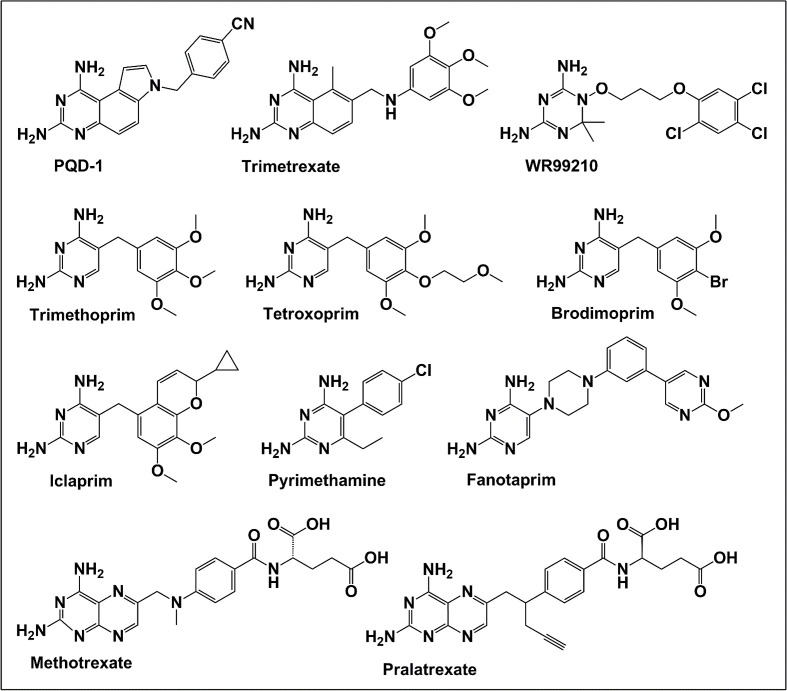
Structures of tested DHFR inhibitors. See Table 1 for references.

To identify a DHFR inhibitor with whole cell activity against *M. abscessus*, three DHFR inhibitors previously shown to be active against *M. tuberculosis* were tested against the reference strain *M. abscessus* subsp. *abscessus* ATCC 19977: the pyrrolo-quinazoline PQD-1 (20), the methyl-quinazoline trimetrexate (21), and the triazine WR99210 (15, 22) (Fig. 1). A selection of additional anti-bacterial, anti-parasitic, and anti-cancer DHFR inhibitors were included in the screen (Fig. 1). The three *M. tuberculosis*-active compounds were active against *M. abscessus*, with PQD-1 being the most potent (MIC_90_, concentration required for 90% growth inhibition = 3 µM) followed by trimetrexate (12 µM) and WR99210 (20 µM) (Table 1). All other DHFR inhibitors, including the anti-bacterial trimethoprim, were inactive (MIC_90_ >100 µM, Table 1).

**TABLE 1:** Activity of DHFR inhibitors against *M. abscessus* ATCC 19977 and its recombinant DHFR enzyme

Drug	Chemical class	Clinical development status	Therapeutic indication	References	MIC_50_ (µM)^*a*^	MIC_90_ (µM)^*a*^	IC_50_ (µM)^*b*^
PQD-1	Quinazoline	NA^*c*^	Anti-malarial	(19, 20)	0.2	3	0.0025
Trimetrexate	Quinazoline	Approved	Anti-fungal	(21)	1.2	12	0.005
WR99210	Triazine	NA	Anti-malarial	(15, 22)	5.6	20	0.016
Trimethoprim	Pyrimidine	Approved	Anti-bacterial	(5)	>100	>100	25
Tetroxoprim	Pyrimidine	Withdrawn	Anti-bacterial	(23)	>100	>100	20
Brodimoprim	Pyrimidine	Withdrawn	Anti-bacterial	(23)	>100	>100	1.7
Iclaprim	Pyrimidine	Phase 3	Anti-bacterial	(24)	36	>100	0.6
Pyrimethamine	Pyrimidine	Approved	Anti-parasitic	(25, 26)	>100	>100	4.7
Fanotaprim	Pyrimidine	Phase 1	Anti-parasitic	(27)	>100	>100	2.5
Methotrexate	Pteridine	Approved	Anti-cancer	(28)	>100	>100	0.016
Pralatrexate	Pteridine	Approved	Anti-cancer	(29)	>100	>100	0.21

^*a*^ MIC_50_ and MIC_90_ values are defined as drug concentrations causing 50% and 90% growth inhibition, respectively, compared to untreated control and are the means of three independent experiments.

^*b*^ IC_50_ values are defined as compound concentrations causing 50% inhibition of *M. abscessus* DHFR enzyme activity compared to untreated control and are the means of three independent experiments.

^*c*^ NA, not applicable.

### PQD-1 inhibits the recombinant *M. abscessus* DHFR enzyme

**Fig 2:**
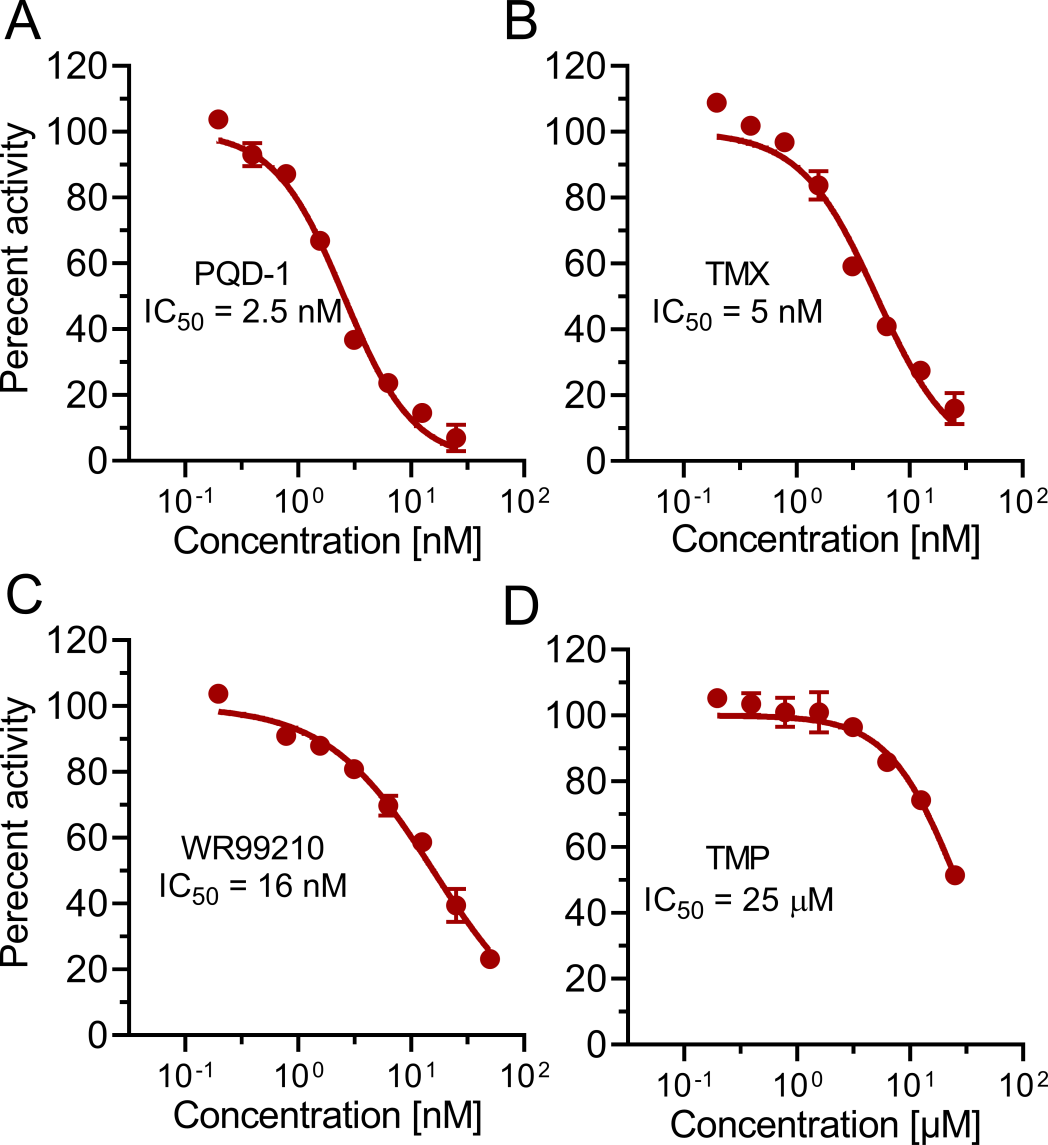
Activity of selected DHFR inhibitors against recombinant *M. abscessus* ATCC 19977 DHFR enzyme. Enzyme inhibition dose-response curves are shown for (**A**) PQD-1, (**B**) trimetrexate (TMX), (**C**) WR99210, and (**D**) trimethoprim (TMP). The data were fitted to a non-linear regression curve using the variable slope model, and the IC_50_ values were calculated using GraphPad Prism 9. Percent activity values are the means of three independently performed experiments, and error bars indicate standard deviations.

To determine whether the DHFR inhibitors are active against *M. abscessus* act via DHFR, the recombinant protein from *M. abscessus* ATCC 19977 was generated and purified (Fig. S1 and S2) for biochemical inhibition assays. PQD-1, trimetrexate, and WR99210 showed low nanomolar IC_50_ (concentration inhibiting half-maximal enzyme activity) against the isolated protein (Fig. 2; Table 1). The other tested anti-bacterial and anti-parasitic DHFR inhibitors were ~1,000-fold less active in the enzymatic assay, mirroring their lack of whole cell activity (Table 1). This included trimethoprim (Fig. 2; Table 1), suggesting that the intrinsic resistance of *M. abscessus* to this antibacterial is due to poor on-target activity. Interestingly, the two anti-cancer pteridines, namely, methotrexate and pralatrexate, had sub-micromolar IC_50_ against the isolated target (Table 1), indicative of good target engagement despite their lack of activity against whole cells (Table 1), pointing to a non-target-related intrinsic resistance mechanism against these molecules, such as poor uptake, active efflux, and/or intrabacterial metabolism.

### PQD-1 interacts favorably with the active site of *M. abscessus* DHFR

**Fig 3:**
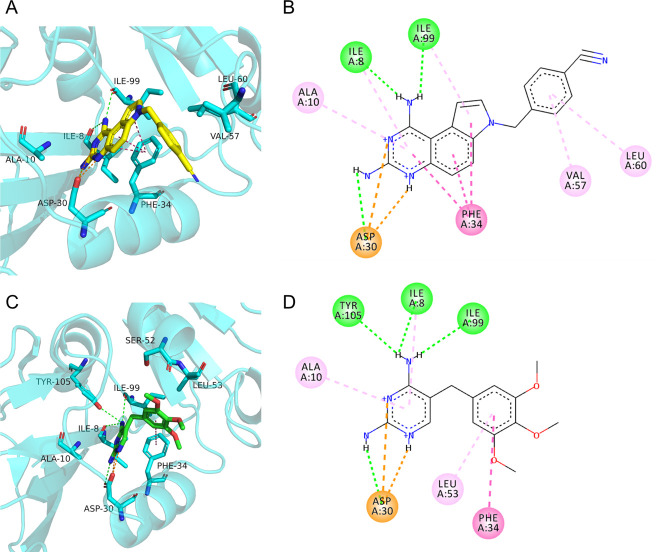
*In silico* model of PQD-1 and trimethoprim binding to *M. abscessus* ATCC 19977 DHFR (PDB code: 7K6C). (**A, C**) Optimized poses of PQD-1 (yellow sticks) and trimethoprim (green sticks) with interactions with DHFR active site residues. DHFR is colored in cyan, with the residues in the binding pockets shown as cyan sticks. (**B, D**) Two-dimensional presentation of the key interactions of PQD-1 (**B**) and trimethoprim (**D**) with their binding pockets shown in (**A, C**). The hydrogen bonds, salt bridges, and hydrophobic and π-π stacking interactions are depicted as green, orange, pink, and warm pink dashed lines, respectively.

To gain structural insight into the binding mode of PQD-1, molecular modeling studies were carried out by docking PQD-1 into the crystal structure of *M. abscessus* DHFR (PDB code: 7K6C). In the conformation with the lowest binding free energy (−7.65 kcal/mol, Fig. 3), the nitrogen atoms of the 2,4-diamino substitution in the pyrimidine ring of PQD-1 form three hydrogen bonds with the oxygen atoms of the active site residues, i.e., Ile8, Ile99, and Asp30 (30). The nitrogen atoms in the pyrimidine heterocycle form favorable charge and salt bridge interactions with Asp30. Furthermore, the aromatic pyrrolo-quinazoline and benzene moieties form hydrophobic interactions with Ala10, Val57, and Leu60 and π-π staking interactions with Phe34 (Fig. 3). Docking of trimethoprim resulted in fewer interactions (Fig. 3) and a weaker docking score of −5.57 kcal/mol, consistent with the lower inhibitory activity of trimethoprim against *M. abscessus* DHFR (Fig. 2; Table 1). As expected, trimethoprim shared PQD-1 binding residues interacting with the diamino-pyrimidine ring, a conserved pharmacophore in DHFR inhibitors (Fig. 1) (4).

### Over-expression of DHFR in *M. abscessus* causes resistance to PQD-1

**Fig 4:**
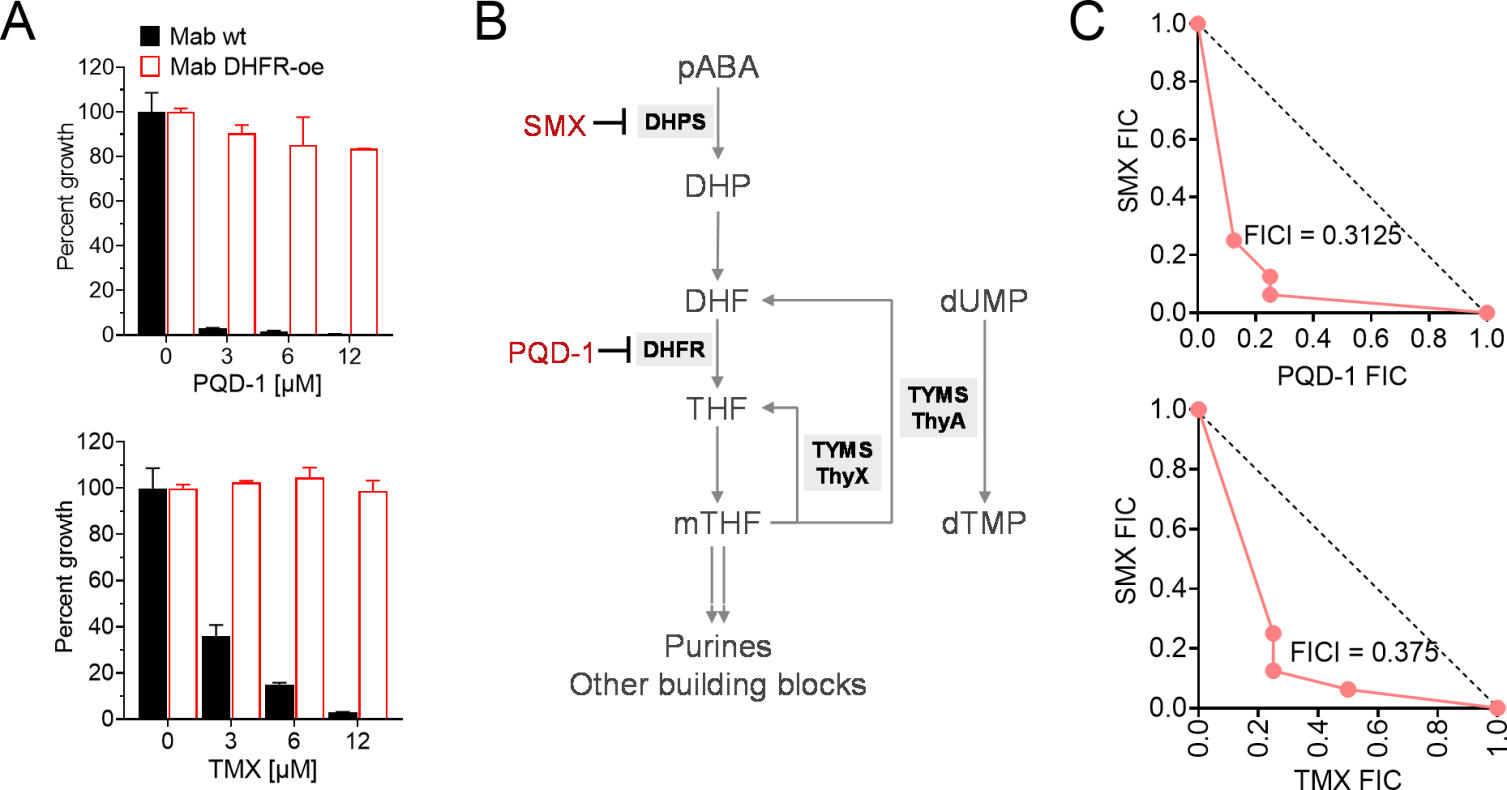
Mechanism of action of PQD-1. (**A**) Effect of DHFR over-expression on PQD-1 susceptibility of *M. abscessus* ATCC 19977. Cultures were treated for 3 days with PQD-1 (top) or TMX (bottom), the second most potent *M. abscessus* inhibitor identified in the initial whole cell screen (Table 1) and confirmed as a biochemical inhibitor of *M. abscessus* DHFR (Fig. 2). Percent growth values are the means of three independently carried out experiments, and error bars indicate standard deviations. (**B**) Schematic of mycobacterial folate pathway and role of thymidylate synthase (TYMS) ThyA. DHPS, inhibited by SMX, converts *p*-aminobenzoic acid (pABA) into dihydropteroate (DHP), which in turn is converted to dihydrofolate (DHF). DHFR, inhibited by PQD-1, reduces DHF to tetrahydrofolate (THF), which is converted into methyl-tetrahydrofolate (mTHF). The TYMS ThyA catalyzes the reductive methylation of deoxyuridine-monophosphate (dUMP) to deoxythymidine-monophosphate (dTMP) utilizing mTHF as the methyl donor and reductant in the reaction, yielding DHF, the substrate of DHFR, as a by-product. ThyX is a second thymidylate synthase catalyzing the reductive methylation of dUMP to dTMP utilizing mTHF only as the methyl donor (hence generating THF instead of DHF) and NADPH as the reductant. The pathway is according to Hajian et al. (31). (**C**) Plot of the fractional inhibitory concentrations (FIC) of PQD-1 (top) and TMX (bottom) versus DHPS inhibitor SMX. The FIC index (FICI), calculated as (MIC_A_^combi^/MIC_A_^alone^) + (MIC_B_^combi^/MIC_B_^alone^), indicates synergy if <0.5. The FICs were calculated based on the MIC_50_ values. The experiments were carried out three times independently in duplicate, and the presented data are one representative example.

To provide functional evidence that PQD-1 exerts its whole cell activity by inhibition of bacterial DHFR, the *M. abscessus* ATCC 19977 *dfrA* gene was over-expressed in *M. abscessus* ATCC 19977 using the *hsp60* promoter carried by plasmid pMV262, and the impact on PQD-1 susceptibility was determined. Over-expression of the DHFR gene caused strong resistance to PQD-1 (Fig. 4A) indicating that PQD-1 acts via the folate pathway and likely inhibits DHFR.

### Spontaneous *M. abscessus* resistance to PQD-1 maps to thymidylate synthase ThyA

We and others have shown that one of the mechanisms of how spontaneous resistance against DHFR inhibitors in *M. tuberculosis* can emerge is via missense mutations in the TYMS ThyA, a folate pathway enzyme downstream of DHFR, rather than missense mutations in the direct target DHFR (32 – 35). TYMS ThyA catalyzes the reductive methylation of dUMP to dTMP utilizing methylenetetrahydrofolate (mTHF) as the methyl donor and reductant in the reaction, yielding dihydrofolate, the substrate of DHFR, as a by-product (Fig. 4B). Missense mutations in TYMS ThyA are thought to reduce enzymatic activity and thus the amount of dihydrofolate produced. This in turn affects the growth inhibitory activity of DHFR inhibitors (35). To determine whether this mechanism of resistance against DHFR inhibitors in *M. tuberculosis* is conserved in *M. abscessus*, we isolated spontaneous resistance mutants. Three independent cultures of *M. abscessus* ATCC 19977 were plated on Middlebrook 7H10 agar containing 33× MIC_90_ (100 µM) of PQD-1, the lowest concentration that suppressed the growth of wild-type colonies on a solid medium. Resistance colonies emerged at a frequency of ~10^−7^/CFU. Three resistant strains from each selection experiment were randomly picked for further analyses. MIC determinations revealed a similar, high-level resistance to PQD-1 (Table 2; Fig. S3). Targeted Sanger sequencing of *thyA* and *dfrA* revealed missense mutations in ThyA in all nine strains and no polymorphisms in *dfrA* (Table 2). Complementation of a PQD-1 resistant *thyA* mutant strain with a copy of wild-type *M. abscessus* ATCC 19977 *thyA* increased susceptibility to PQD-1 and thus partially reverted the phenotype (Fig. S4). These results suggest that spontaneous resistance against PQD-1 can emerge via missense mutation in *thyA*, similar to what was observed for DHFR inhibitors active against *M. tuberculosis*.

**TABLE 2:** Characterization of spontaneous PQD-1 resistant *M. abscessus* ATCC 19977 strains^*a*^

Exp.#	Strain	Polymorphism (nt/aa)		MIC_90_ (µM)		
		dfrA	thyA	PQD-1	TMX	CLR
	wt	wt	wt	3	12	1.6
1	PQD^R^1.1	wt	C440A/P147Q	50	100	1.6
	PQD^R^1.2	wt	C440A/P147Q	50	100	1.6
	PQD^R^1.3	wt	G300C/Q100H	>50	>100	1.6
2	PQD^R^2.1	wt	G451C/A151P	25	100	1.6
	PQD^R^2.2	wt	C440A/P147Q	50	100	1.6
	PQD^R^2.3	wt	C440A/P147Q	50	100	1.6
3	PQD^R^3.1	wt	C440A/P147Q	50	100	1.6
	PQD^R^3.2	wt	C440A/P147Q	50	100	1.6
	PQD^R^3.3	wt	C440A/P147Q	50	100	1.6

^*a*^ Exp.#, independent selection experiments with independently grown culture batches; wt, wild type; PQD^R^, PQD-1 resistant strain; TMX, trimetrexate; CLR, clarithromycin; nt/aa, nucleotide sequence polymorphism and associated amino acid substitution. MIC_90_, drug concentrations causing 90% growth inhibition compared to untreated control. The MIC experiments were carried out three times independently, and the mean values are shown (see Fig. S3 for dose-response curves).

In the past decade, two non-canonical folate enzymes, RibD (a second functional DHFR) and ThyX (a second thymidylate synthase, Fig. 4B) were found to be involved in the mechanisms of action and resistance of antifolates in *M. tuberculosis* (31, 34). The *M. abscessus* genome harbors potential homologs of these genes (MAB_2976, MAB_3085 c) (10). To determine whether the PQD-1 resistant *thyA* mutant strains harbor additional mutations in these loci, which may contribute to their PQD-1 resistance, the nine strains were subjected to whole genome sequencing (Table S1). Additional consistent polymorphisms were not detected. Importantly, no polymorphisms were detected in the *ribD* or *thyX* coding or upstream sequences (Table S1). This suggests that the observed reduced PDQ-1 susceptibility in the nine resistant strains is solely due to the mutations in *thyA*. It is to be noted that the result of our PQD-1 resistance analysis does not exclude the possibility that characterization of a larger number of PQD-1-resistant *M. abscessus* strains may reveal additional resistance mechanisms due to mutations in *ribD*, *thyX*, or other genes.

### PQD-1 analogs reveal a dynamic SAR against *M. abscessus*

To explore the SAR around the PQD-1 scaffold, nine analogs were retrieved from the Merck & Co., Inc., Rahway, NJ, USA, compound archive. MIC against *M. abscessus* and IC_50_ against its recombinant DHFR enzyme were determined. The analogs, which mostly retained the pyrrolo-quinazoline moiety of PQD-1 and carried modifications in the eastern part of the scaffold (Table 3), showed a range of potency (Table 3) suggesting dynamic SAR and thus suitability of PQD-1 as a starting point for chemical optimization. Thus, the liabilities of PQD-1, briefly inadequate metabolic stability, and poor selectivity towards the human enzyme (19), can now be addressed in a lead optimization program aimed at improving pharmacokinetic properties and introducing specificity for the mycobacterial enzyme. Whether the liabilities can be removed while retaining on-target activity remains to be determined.

**TABLE 3:** Dynamic SAR of PQD-1 analogs against *M. abscessus* ATCC 19977 and its recombinant DHFR enzyme

Compound	Structure	MIC_50_ ^*a*^ (µM)	MIC_90_ ^*a*^ (µM)	IC_50_ ^*b*^ (µM)
PQD-1	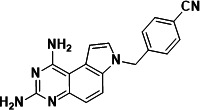	0.2	3	0.0025
PQD-2	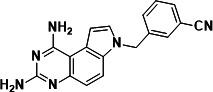	0.7	5	0.0034
PQD-3	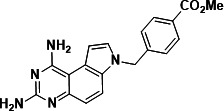	0.7	6.5	0.015
PQD-4	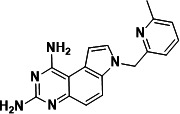	6	12	0.015
PQD-5	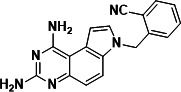	2	20	0.016

PQD-6	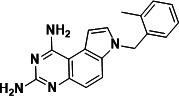	6	20	0.015
PQD-7	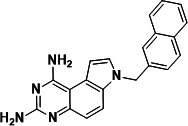	6	>25	0.015
PQD-8	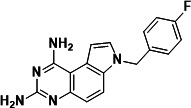	6	20	0.02
PQD-9	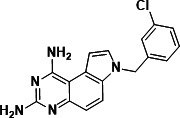	6	20	0.03
PQD-10	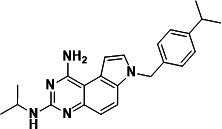	15	20	4.1

^*a*^ MIC_50_ and MIC_90_ values are defined as compound concentrations causing 50% and 90% growth inhibition, respectively, compared to untreated control and are the means of three independent experiments.

^*b*^ IC_50_ values are defined as compound concentrations causing 50% inhibition of *M. abscessus* DHFR enzyme activity compared to untreated control and are the means of three independent experiments.

### PQD-1 synergizes with the dihydropteroate synthase inhibitor sulfamethoxazole in *M. abscessus*

In other bacteria, DHFR inhibitors typically synergize with sulfonamide inhibitors of DHPS (5). Growth inhibition experiments showed that sulfamethoxazole alone is active against *M. abscessus* whole cells, with an MIC_50_ of 12 µM (MIC_90_ >100 µM). To determine whether PQD-1 and sulfamethoxazole synergize against *M. abscessus*, checkerboard growth inhibition experiments were carried out, revealing a fractional inhibitory concentration index (FICI) of 0.3, indicative of synergy between PQD-1 and sulfamethoxazole (Fig. 4C). This result paves the way for the development of a Bactrim-like synergistic drug combination for the treatment of *M. abscessus* infections.

### PQD-1 is active against *M. abscessus* subspecies reference strains and a panel of clinical isolates

To determine whether the attractive activity of PQD-1 against the type strain *M. abscessus* subsp. *abscessus* ATCC 19977 was retained against *M. abscessus* subspecies, growth inhibition activities were measured against *M. abscessus* subsp. *massiliense* and *bolletii* reference strains and a panel of clinical isolates. PQD-1 retained activity against this strain collection indicating that PQD-1 is broadly active against the *M. abscessus* complex and providing epidemiological validation of the DHFR-PQD-1 target-lead pair (Table 4).

**TABLE 4:** Activity of PQD-1 against *M. abscessus* subspecies reference strains and clinical isolates

*M. abscessus* strain	*erm41* sequevar^*a*^	MIC_90_ (µM)^*b*^		
		PQD-1	TMX	CLR
Reference strains				
Subsp. *abscessus* ATCC 19977	T28	3	12.5	3
Subsp. *bolletii* CCUG 50,184T	T28	12.5	25	6
Subsp. *massiliense* CCUG 48,898T	Deletion	3	12.5	1.6
Clinical isolates				
Subsp. *abscessus* Bamboo	C28	6	12.5	0.8
Subsp. *abscessus* K21	C28	3	6	0.8
Subsp. *abscessus* M9	T28	3	12.5	6
Subsp. *abscessus* M199	T28	6	12.5	6
Subsp. *abscessus* M337	T28	3	12.5	6
Subsp. *abscessus* M404	C28	3	6	0.8
Subsp. *abscessus* M422	T28	3	6	3
Subsp. *bolletii* M232	T28	3	6	6
Subsp. *bolletii* M506	C28	6	12.5	3
Subsp. *massiliense* M111	Deletion	2	6	1.6

^*a*^ *erm41*, ribosome methylase. T28 sequevars confer inducible clarithromycin (CLR) resistance. C28 and “deletion” sequevars are CLR-sensitive (36).

^*b*^ MIC_90_ values are defined as drug concentrations causing 90% growth inhibition compared to untreated control and are the means of two independent experiments. TMX, trimetrexate; CLR, clarithromycin.

In summary, we have shown that *M. abscessus* DHFR is a vulnerable target and that the intrinsic resistance of *M. abscessus* to trimethoprim is due to poor activity against the *M. abscessus* DHFR enzyme. Biochemical data, complemented by docking and genetic studies, indicate that PQD-1 exerts whole cell anti-*M*. *abscessus* activity by inhibiting DHFR, thus pharmacologically validating the target. Screening against a panel of *M. abscessus* strains shows that target vulnerability is epidemiologically conserved, confirming DHFR/PQD-1 as an attractive target-lead couple. Dynamic SAR around the PQD-1 scaffold and synergy with the DHPS inhibitor sulfamethoxazole support a dual targeting approach of the folate pathway in *M. abscessus*, similar to the broadly successful Bactrim antibiotic.

## MATERIALS AND METHODS

### Bacterial strains, media, and culture conditions

*M. abscessus* subsp. *abscessus* ATCC 19977 was purchased from the American Type Culture Collection. *M. abscessus* subsp. *bolletii* CCUG 50,184T and *M. abscessus* subsp. *massiliense* CCUG 48,898T was purchased from the Culture Collection University of Goteborg. *M. abscessus* subsp. *abscessus* Bamboo was provided by Wei Chang Huang (Taichung Veterans General Hospital, Taichung, Taiwan). *M. abscessus* subsp. *abscessus* K21 was provided by Sung Jae Shin (Department of Microbiology, Yonsei University College of Medicine, Seoul, South Korea) and Won-Jung Koh (Division of Pulmonary and Critical Care Medicine, Samsung Medical Center, Seoul, South Korea). The clinical M isolates of *M. abscessus* were provided by Jeanette W. P. Teo (Department of Laboratory Medicine, National University Hospital of Singapore).

*M. abscessus* strains were grown in Middlebrook 7H9 broth (BD Difco) supplemented with 0.05% (vol/vol) Tween 80 (Sigma), 0.2% (vol/vol) glycerol (Fisher Scientific), and 10% (vol/vol) Middlebrook albumin-dextrose-catalase (BD Difco) at 37°C with agitation at 90 rpm (INFORS HT Multitron Pro). For determination of CFU, bacterial cultures were spread onto Middlebrook 7H10 agar (BD Difco) supplemented with 10% (vol/vol) Middlebrook oleic acid-albumin-dextrose-catalase and 0.2% glycerol and grown at 37°C. When appropriate, agar was supplemented with PQD-1 for the isolation of resistant mutants or 400-µg/mL kanamycin sulfate for the selection of transformed bacteria in the over-expression and complementation experiments. *Escherichia coli* strains TOP10 and BL21 (DE3) were used for propagation of plasmids and expression of recombinant DHFR, respectively, and were cultured in Luria-Bertani (LB) broth or on LB agar (BD Difco) without or with 50-µg/L kanamycin sulfate.

### Drugs and chemicals

PQD-1, trimetrexate, tetroxoprim, brodimoprim, iclaprim, fanotaprim, and WR99210 were purchased from MedChemExpress LLC. Trimethoprim, pyrimethamine, pralatrexate, methotrexate, sulfamethoxazole, kanamycin sulfate, and clarithromycin were purchased from Sigma-Aldrich Inc. PQD-1 analogs were from Merck & Co., Inc., Rahway, NJ, USA, compound archive. All chemicals were dissolved in dimethyl sulfoxide [(DMSO) Sigma-Aldrich Inc.] except kanamycin sulfate, which was dissolved to 50 mg/mL in deionized water and sterilized using 0.2-µm Minisart high-flow syringe filters (Sartorius). The final DMSO concentration in bacterial culture assays was 1%.

### Growth inhibition assay

Dose-response growth inhibition was measured as described previously (37). Briefly, for each inhibitor a two-fold dilution series starting at the desired highest concentration was dispensed onto flat-bottom 96-well plates using a D300e Digital Dispenser (Tecan). A 200-µL mid-log-phase culture of OD_600 nm_ = 0.05 was dispensed to each drug-containing well. Untreated control wells were included on each plate. Plates were sealed with parafilm, stored in boxes with wet paper towels, and incubated for 3 days at 37°C with shaking. Growth was monitored by measuring OD_600 nm_ using a Tecan Infinite 200 Pro microplate reader (Tecan). Day 0 values were subtracted from the corresponding end-point values, and percentage growth was calculated by dividing the growth value in the drug-containing well by the average growth value of the untreated control wells and multiplying by 100. Dose-response curves were generated by plotting drug concentrations versus percentage growth using GraphPad Prism 9.

### Production of recombinant DHFR

Production of recombinant DHFR was carried out by Wuxi Biologics Co., Ltd., Shanghai, China. *E. coli* codon-optimized sequence of *M. abscessus* DHFR (MAB_3090 c) was cloned into the expression vector pET28a at the *Nco*I and *Xho*I restriction sites to express DHFR with an N-terminal His6-tag. The expression vector was transformed by adding 50 ng of DNA to BL21(DE3) *E. coli* competent cells followed by heat shock. Bacterial cultures were expanded in LB medium and incubated at 37˚C until OD_600 nm_ reached 0.6 ~ 0.8. Isopropyl β- d-1-thiogalactopyranoside (IPTG) at 0.2 mM was introduced for induction, and cultures were kept shaking at 18˚C overnight. The cells were centrifuged at 7,000 × *g* for 15 min, resuspended in 50-mM Tris-HCl, 150-mM NaCl, 20-mM imidazole, 1-mM tris(2-carboxyethyl)phosphine (TCEP), and 10% glycerol, at pH 7.5, and lysed by high-pressure homogenization. DHFR protein was purified with Ni-NTA resin and eluted in 20-mM Tris-HCl, 300-mM NaCl, 250-mM imidazole, 0.5-mM TCEP, and 10% glycerol, at pH 7.5. Then, the buffer was exchanged into a final storage buffer with 20-mM Tris-HCl, 150-mM NaCl, 0.5-mM TCEP, and 10% glycerol, at pH 7.5. The concentration of the purified DHFR was measured by the Bradford method. Non-reduced and reduced [100-mM dithiothreitol (DTT)] SDS-PAGE was performed with Bio-Rad equipment. Non-reduced intact mass data were obtained on an Agilent electrospray ionization time-of-flight (ESI-TOF) mass spectrometer (6230) by injecting 1.5 µg of the final product. Further analysis was carried out with the Agilent BioConfirm software.

### DHFR inhibition assay

Enzyme inhibition assays were performed according to the manufacturer’s instructions using a DHFR assay kit purchased from Sigma-Aldrich (CS0340, Sigma-Aldrich Inc.values are defined as drug concentrations ), with minor modifications. In brief, 120-µM NADPH was mixed with 0.6 µg/mL *M*. *abscessus* DHFR, and 50  µL of the resulting mix was dispensed onto 96-well plates. For each drug/compound, a twofold dilution series starting at twice the desired highest concentration was dispensed onto the 96-well plates using a Tecan D300e Digital Dispenser. The reaction was started by adding 50 µL of 100-µM DHF to each well to give a final volume of 100  µL per well with final NADPH, DHFR, DHF, and DMSO concentrations of 60 µM, 0.3 µg/mL, 50 µM, and 0.25%, respectively. For each experiment, drug-free and enzyme-free controls were included in which DMSO was added to the reaction mix instead of test drugs and DHFR, respectively. All the reagents were prepared with 1 × DHFR assay buffer provided by the kit. The reaction progress was monitored by measuring the decrease in the absorbance (340 nm) at 30-second intervals for 10 minutes at room temperature using a Tecan Infinite 200 Pro microplate reader (Tecan). A graph of absorbance values versus time was plotted, and the slope of the graph over the linear range was taken to represent the velocity of the reaction. Percentage activity was calculated by dividing the slope of the inhibited enzyme by the average slope of the uninhibited enzyme and multiplying by 100. The IC_50_ values were calculated by fitting the data to a non-linear regression sigmoidal dose-response curves (variable slope) using GraphPad Prism 9.5.1.

### *In silico* docking

*In silico* docking was carried out by BOC Sciences Inc., NY, USA. AutoDock-GPU, an OpenCL-accelerated version of AutoDock4 running on GPUs, was used for molecular docking experiments (38). The crystal structure of *M. abscessus* DHFR (PDB code: 7K6C) was used as a receptor. AutoDock used the Lamarckian genetic algorithm and the empirical free energy scoring function and typically provided reproducible docking results for ligands. During the docking process, the coordinate files of protein and ligand were created, which included polar hydrogen atoms, partial charges, atom types, and information on the articulation of flexible molecules. The grid parameter file of the binding pocket was created with the grid center 1.786, 1.766, 58.555 (x, y, z in Å), and dimensions 30 × 30 × 30 Å referring to the binding sites of the ligand in 7K6C. A conformation search algorithm was used to explore the conformational states of a flexible ligand, using the grid maps to evaluate ligand-protein interactions at each point in the docking simulation. The docking results were clustered to identify similar conformations. The conformation with the lowest binding free energy was identified as the most probable conformation. The interactions between the protein-ligand complex were mapped by PyMOL.

### Over-expression of DHFR in *M. abscessus*

Wild-type *M. abscessus* ATCC19977 *dfrA* was cloned into plasmid pMV262 (39) to overexpress the gene under the control of the constitutive *hsp60* promoter as described previously (16). The coding sequence of *dfrA* was PCR amplified (Phusion high-fidelity DNA polymerase, Fisher Scientific) from *M. abscessus* ATCC19977 genomic DNA with primers CCG **GGATCC** ATGACGGGAACCATCGGG (*Bam*HI, forward) and CCG **GAATTC** TCACCCGTCGACTTTCCG (*Eco*RI, reverse), inserted in frame (*Bam*HI-*Eco*RI), and transformed into *E. coli* TOP10. Positive clones were identified by PCR after which the integrity of the construct was confirmed by Sanger sequencing (Azenta Life Sciences). The purified plasmid DNA was electroporated into *M. abscessus* ATCC 19977 as previously described (40). *M. abscessus* transformants were selected on 7H10 agar containing 400-µg/mL kanamycin.

### Selection and analysis of spontaneous resistant mutants

The mutant selection was carried out as previously described (37). In brief, bacterial inocula (2  ×  10^8^) from mid-log cultures of *M. abscessus* ATCC 19977 were spread on 7H10 agar containing 33 x MIC_90_ of PQD-1, the lowest concentration that suppressed the growth of wild-type colonies and grown at 37°C for 10 days. Putative-resistant colonies were picked, inoculated into 7H9 broth, expanded to mid-log phase, and stored with 10% glycerol at –80°C until used for subsequent studies. The PQD-1 resistance levels of mutant strains were measured by generating dose-response curves with the parent (wild type) strain as a control. Resistance mutations were identified by Sanger sequencing. Genomic DNA was extracted using the phenol-chloroform method as described previously (32). Targeted sequencing of *dfrA* (MAB_3090 c) and *thyA* (MAB_3091 c) genes was performed by Genewiz Inc. (South Plainfield, NJ, USA) using forward-1 CGGTCAGAACGAGCAGCAC, forward-2 GTAGCCGCCAGGGAATGG, and reverse CGACGCTCAGCTGACCACG primers. Whole genome re-sequencing and bioinformatics analyses were carried out by Novogene Corporation Inc. (Sacramento, CA, USA) as described previously (37). Sequencing data are available from the authors upon request. The GenBank accession number for the sequence of the parent strain *M. abscessus* ATCC 19977 is CU458896.1.

### Complementation of ThyA mutants in *M. abscessus*

The wild-type *thyA* (MAB_3091 c) gene was cloned into plasmid pMV262 to be expressed under the control of the *hsp60* promoter. The coding region of *thyA* was PCR amplified (Phusion high-fidelity DNA polymerase, Fisher Scientific) from *M. abscessus* ATCC19977 genomic DNA with primers forward (*Eco*RI) CGC **GAATTC** GTGTCTCTTCCCACTCCCTACG and reverse (*Hind*III) GCG **AAGCTT** TCATACCGCGACCGGCG, inserted in frame (*Eco*RI-*Hind*III), and transformed into *E. coli* TOP10. Positive clones were identified by PCR, sequence integrity was confirmed by targeted sequencing (Genewiz Inc., South Plainfield, NJ, USA), and the plasmid DNA was electroporated to *M. abscessus* PQD^R^1.3 harboring the *thyA* mutation Q100H. Transformant *M. abscessus* was selected on 7H10 agar containing 400  µg/mL kanamycin.

### *In vitro* synergy assay

The synergistic activity between PQD-1 or TMX and SMX was measured using the checkerboard titration assay (41). In the combination, 11 concentrations of PQD-1 or TMX in twofold dilution were tested for synergy against eight concentrations of SMX, dispensed using the Tecan D300e Digital Dispenser (Tecan). Inoculum preparation, incubation, and measurement of growth inhibition were done as described in the section “Growth inhibition assay.” The FICI was used to analyze the results. FICI was derived using concentrations at which 50% inhibition of growth was observed. FICI was calculated as (MIC_A_^combi^/MIC_A_^alone^) + (MIC_B_^combi^/MIC_B_^alone^) and indicates synergy if <0.5.

## SUPPLEMENTAL MATERIAL

Fig. S1-S4, Table S1 aac.00717-23-s0001.pdf Supplemental Material.

ASM does not own the copyrights to Supplemental Material that may be linked to, or accessed through, an article. The authors have granted ASM a non-exclusive, world-wide license to publish the Supplemental Material files. Please contact the corresponding author directly for reuse.
