# Supplementary material for: Pharmacological validation of dihydrofolate reductase as a drug target in Mycobacterium abscessus
Source: Antimicrob Agents Chemother. 2023 Nov 29;68(1):e00717-23. doi: 10.1128/aac.00717-23 (PMC10777855; doi:10.1128/aac.00717-23)
Supplement: Fig. S1-S4, Table S1 — Supplemental Material. [file aac.00717-23-s0001.pdf]

Supplemental materials

Pharmacological validation of dihydrofolate reductase as a drug target in *Mycobacterium abscessus*

Wassihun Wedajo Aragaw,[a] Dereje A. Negatu,[a] Christopher J. Bungard,[b] Véronique Dartois,[a,c] Abdellatif El Marrouni,[b] Elliott B. Nickbarg,[d] David B. Olsen,[b] Ralf Warrass,[e] and Thomas Dick[a, c, f #]

[a] Center for Discovery and Innovation, Hackensack Meridian Health, Nutley, New Jersey, USA

[b] Merck & Co., Inc., West Point, Pennsylvania, USA

[c] Department of Medical Sciences, Hackensack Meridian School of Medicine, Nutley, New Jersey, USA

[d] Merck & Co., Inc., Boston, Massachusetts, USA

[e] MSD Animal Health Innovation GmbH, Zur Propstei, 55270 Schwabenheim, Germany

[f] Department of Microbiology and Immunology, Georgetown University, Washington, DC, USA

Running Title: DHFR a target for *M. abscessus*

Keywords: Non-tuberculous mycobacteria, NTM, folate pathway, synergy, DHFR, DHPS, ThyA

[#]Address correspondence to Thomas Dick: thomas.dick.cdi@gmail.com

Supplemental figures and table

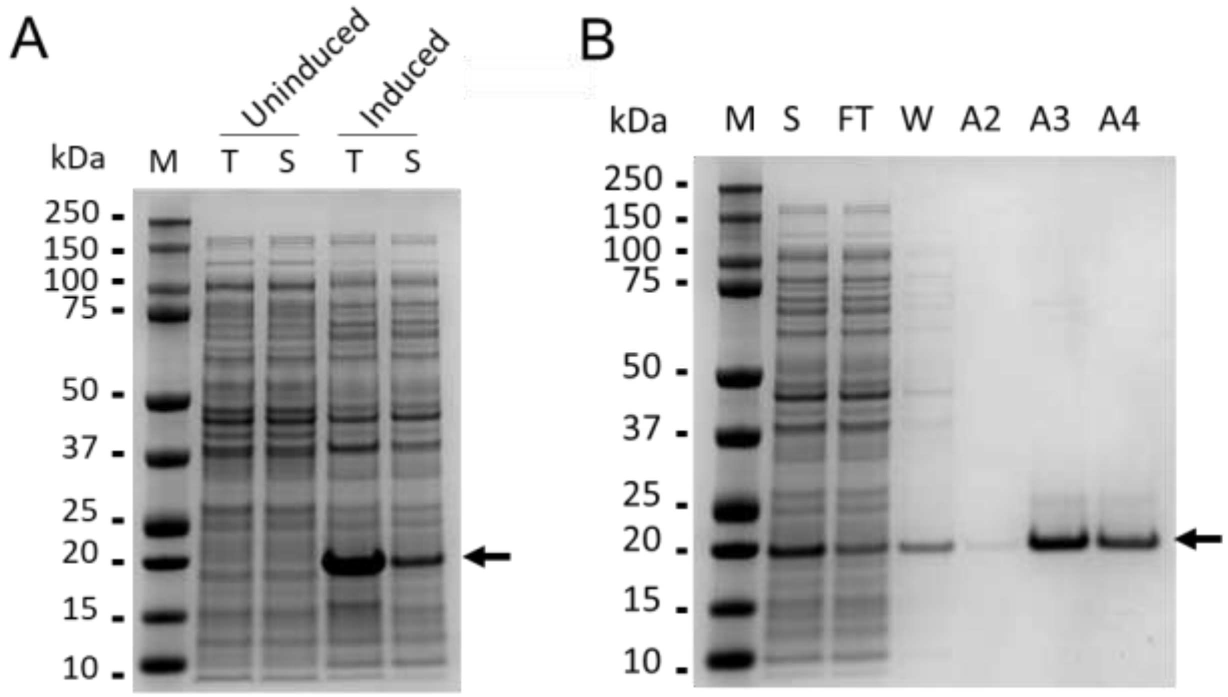

Fig. S1. Purification of His-tagged *M. abscessus* DHFR. (A) SDS-PAGE of the recombinant protein upon IPTG induction. The lysate (T) and the supernatant (S) from the uninduced and induced cultures were loaded to gel lanes together with a molecular weight marker (M). *M. abscessus* DHFR was partially soluble. (B) SDS-PAGE of purified protein fractions. Gel lanes were loaded with molecular weight marker (M), supernatant (S), flow-through (FT), wash (W) and eluted fractions (A2, A3 and A4).

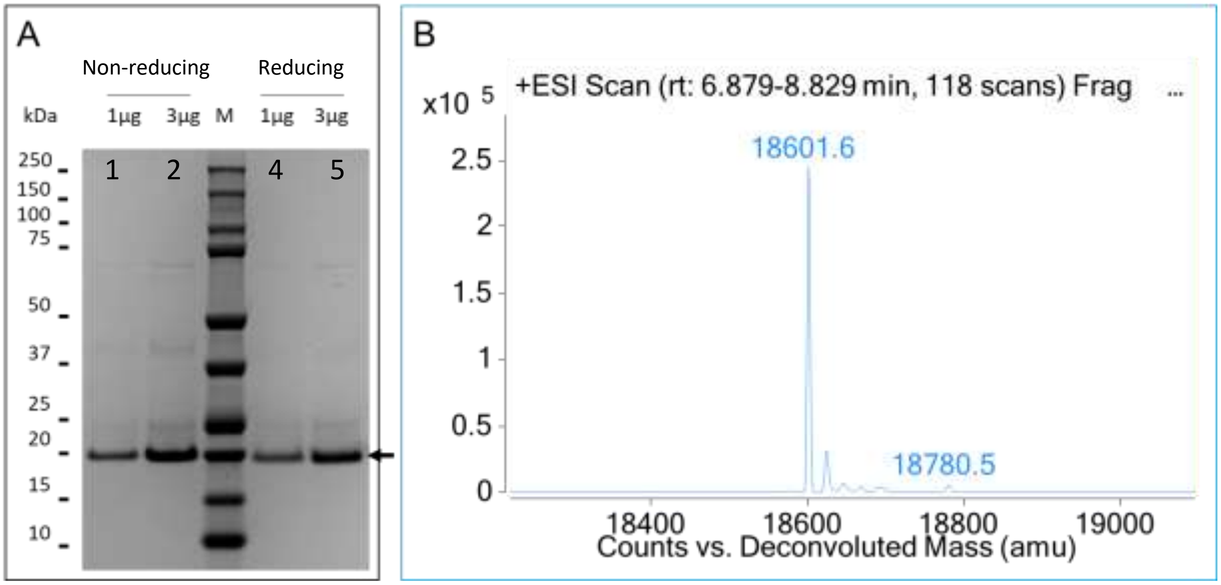

Fig. S2. Quality analysis of the purified *M. abscessus* DHFR. (A) SDS-PAGE analysis in non-reducing (lanes 1 and 2) and reducing conditions (lanes 4 and 5). 1 µg or 3 µg of purified protein was loaded. Band sizes (kDa) of the molecular weight marker (M) are indicated on the left. (B) High pressure liquid chromatography coupled to mass spectrometry (LC-MS) analysis and intact mass detection. Shown are the deconvoluted positive mode electrospray ionization (+ESI) mass spectra. Theoretical mass (Da) is 18601.2 and measured mass (Da) is 18601.6.

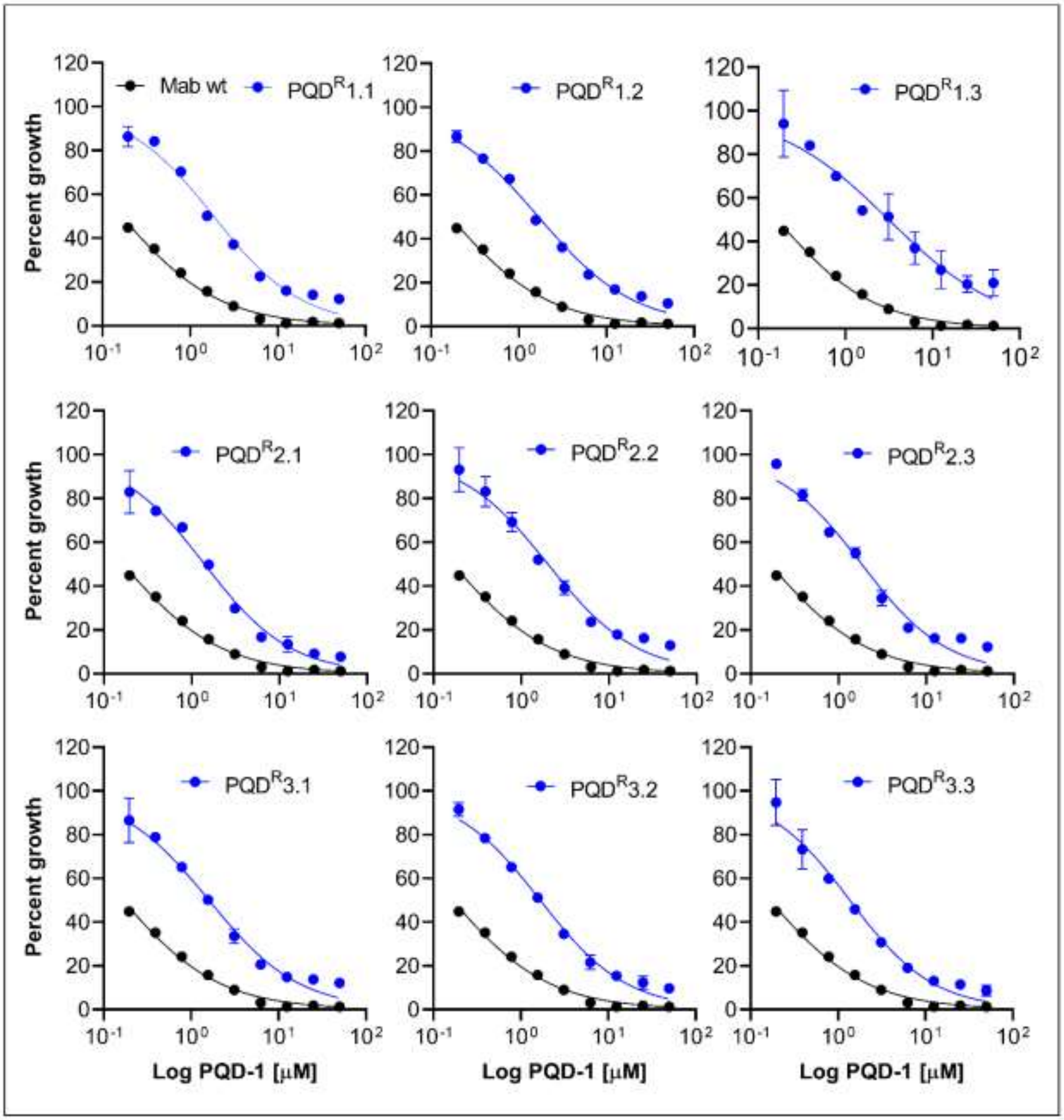

Fig. S3. PQD-1 growth inhibition dose-response curves for PQD-1 resistant *M. abscessus* ATCC 19977 strains. See Table 2 for MIC_{90} and genotypes. The experiments were carried out three times independently and mean values with standard deviations are shown.

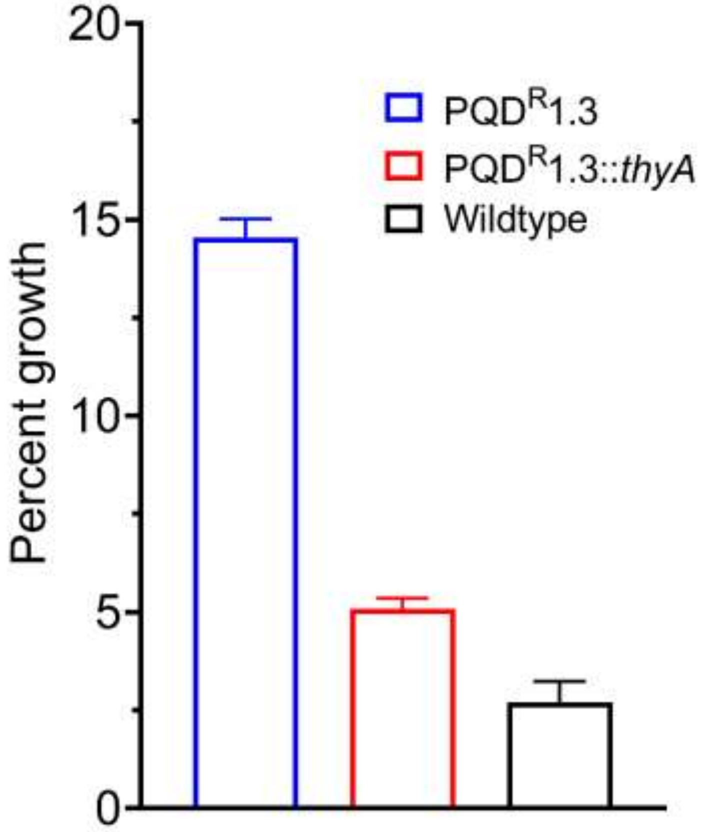

Fig. S4. Complementation of PQD-1 resistant *M. abscessus* with wildtype *thyA*. PQD-1 resistant *M. abscessus* ATCC 19977 harboring a Q100H mutation in ThyA (strain PQD^{R}1.3, Table 2) was transformed with plasmid pMV262 (2) carrying wildtype *thyA* constitutively expressed from the *hsp60* promoter (PQD^{R}1.3::*thyA*). Wildtype *M. abscessus* was used as a control. Cultures were treated with 33x MIC$_{90}$ of PQD-1 (100 µM, Table 1) for 3 days and growth was measured by OD$_{600}$ determination. The experiments were carried out three times independently, and the results are represented as mean values with error bars indicating standard deviations.

TABLE S1. DNA sequence polymorphisms uncovered by whole genome re-sequencing of PQD-1 resistant *thyA* mutant *M. abscessus* ATCC 19977 strains PQD^{R}1.1 to PQD^{R}3.3.

		Polymorphism (nt/aa)				
**Exp.**	**Strain**	***dfrA*** **(MAB_3090c)**	***thyA*** **(MAB_3091c)**	***thyX*** **(MAB_3085c)***	***ribD*** **(MAB_2976)**[*]	**Additional genes**[#] **(gene name: polymorphism)**
	wt	wt	wt	wt	wt	wt
1	PQD^{R}1.1	wt	C440A/P147Q	wt	wt	wt
	PQD^{R}1.2	wt	C440A/P147Q	wt	wt	MAB_2418: T(-58)A, upstream<br>MAB_2885: 300_332Del/100_111Del, non-fs
	PQD^{R}1.3	wt	G300C/Q100H	wt	wt	wt
2	PQD^{R}2.1	wt	G451C/A151P	wt	wt	wt
	PQD^{R}2.2	wt	C440A/P147Q	wt	wt	MAB_4851c: A49C/ T17P, ms
	PQD^{R}2.3	wt	C440A/P147Q	wt	wt	wt
3	PQD^{R}3.1	wt	C440A/P147Q	wt	wt	wt
	PQD^{R}3.2	wt	C440A/P147Q	wt	wt	MAB_4851c: A49C/ T17P, ms
	PQD^{R}3.3	wt	C440A/P147Q	wt	wt	wt

Exp., independently grown culture batches; wt, wild type; PQD^{R}, PQD-1 resistant strain. Polymorphisms within the *thyA* gene were first identified by Sanger sequencing and are presented in Table 2.

[*]Including coding sequence and 500 bp upstream region.

[#] Del, deletion; fs, frameshift; ms, missense mutation.

Supplemental references

1. Robert X, Gouet P. 2014. Deciphering key features in protein structures with the new ENDscript server. Nucleic acids research 42:W320-W324.
2. Stover C, De La Cruz V, Fuerst T, Burlein J, Benson L, Bennett L, Bansal G, Young J, Lee M, Hatfull G. 1991. New use of BCG for recombinant vaccines. Nature 351:456-460.
